# Age- and sex-specific profiles of temporal fasting plasma glucose variability in a population undergoing routine health screening

**DOI:** 10.1186/s12889-021-10367-x

**Published:** 2021-02-09

**Authors:** Agyei Helena Lartey, Xiaona Li, Zhongqi Li, Qun Zhang, Jianming Wang

**Affiliations:** 1 Department of Epidemiology, Center for Global Health, School of Public Health, Nanjing Medical University, Nanjing, 211166 China; 2 Department of Health Management, Center for Global Health, School of Public Health, Nanjing Medical University, Nanjing, 211166 China; 3 Health Management Center, the First Affiliated Hospital of Nanjing Medical University, Nanjing, 210029 China

**Keywords:** Fasting plasma glucose, Diabetes, Variability, Temporal, Coefficient of variation

## Abstract

**Background:**

Fasting plasma glucose (FPG) variability is a significant predictor of mortality, especially in patients with poor glycemic control. This study aimed to explore the temporal age- and sex-specific profiles of temporal FPG variability in a Chinese population undergoing routine health screening and to guide the development of targeted public health interventions for the prevention and control of diabetes.

**Methods:**

In this cross-sectional study, we used a general linear model to compare differences in temporal FPG values between sexes and across age groups in 101,886 Nanjing residents who underwent a routine physical health examination at the Health Management Center, the First Affiliated Hospital of Nanjing Medical University, in 2018. The variability of FPG as a function of time, age, and sex, independently and in combination, was analyzed.

**Results:**

The participants included 57,455 (56.4%) males and 44,431 (43.6%) females, with a mean ± SD age of 42.8 ± 15.0 years. The average ± SD FPG level was 5.5 ± 1.1 mmol/L. The monthly variation contributed to 22% of the overall FPG variability. A significant main effect for the age group was observed (F = 7.39, *P* < 0.05), with an excellent fitting effect (Eta-squared =0.15). The variability of FPG showed sex differences in the percentage difference of the coefficient of variation, which was 34.1% higher in males than females. There were significant interaction effects for month*age*sex and day*age*sex.

**Conclusions:**

Temporal variability in FPG is evident in the general Chinese population and is affected by both age and sex. To avoid complications associated with FPG variability, interventions should be directed at females and males at specific ages for optimal control of FPG variability and to reduce the risk of diabetes and cardiovascular events.

**Supplementary Information:**

The online version contains supplementary material available at 10.1186/s12889-021-10367-x.

## Background

Human health outcomes are unequally distributed throughout populations [1]. Due to differences among population groups subdivided according to age and other demographic characteristics over time, there is a need for epidemiological studies to evaluate observed trends, plan for health care services, and launch interventions to improve health [2, 3].

A fasting plasma glucose (FPG) test is usually used in the diagnosis of diabetes. Plasma glucose levels are controlled by the release of insulin to ensure a dynamic equilibrium (homeostasis). Glycemic or glucose variability makes it difficult to achieve good glycemic control. Generally, blood sugar levels between 70 mg/dL (3.9 mmol/l) and 100 mg/dL l (5.6 mmol/) are considered normal. It is evident that glucose variability (GV) plays a role in the development of diabetic complications, particularly cardiovascular events [4, 5]. FPG variability is an important predictor of mortality, especially in patients with poor glycemic control [6]. Elevated blood glucose was estimated to cause 3.4 million deaths in 2004, equivalent to 5.8% of all deaths [7, 8]. Reduced glycemic exposure affected the development and progression of microvascular complications of diabetes were reported in the Diabetes Control and Complications Trial [9] and the UK Prospective Diabetes Study [10]. Hypoglycemia has been linked to cardiovascular events and increased dementia [11, 12]. Impaired glucose tolerance and impaired fasting glycemia are risks for the future development of diabetes and cardiovascular disease. Generally, lowering the glucose level is essential in the treatment of diabetes, with apparent beneficial effects on microvascular and macrovascular outcomes. Nevertheless, patients with the same glycosylated hemoglobin levels and mean glucose values can have markedly different daily glucose variances [13].

Time-dependent FPG variation, as represented by the coefficient of variation (CV), could predict mortality in subsequent all-cause, expanded or nonexpanded cardiovascular disease-related mortality independent of mean FPG, renal function, mean glycated hemoglobin A1C (HbA1C), HbA1C variation, and other risk factors in patients with type 2 diabetes, suggesting that GV may be a valuable clinical biomarker in the management of patients [5, 14]. Day-to-day self-monitored FPG variability is found to be related to the risk of hypoglycemia in insulin-treated patients with diabetes [15].

To modify risk factors related to impaired glycemic control and diabetes, there is a need to identify populations at risk of developing impaired fasting glucose (IFG) and diabetes and to find measures to curb the progression. An uncontrolled and progressive increase in FPG can predict impending IFG and diabetes. In the current study, we explored the variability of FPG among a general population in eastern China to reveal the blood glucose distribution by age and sex for use in a targeted public health intervention.

## Methods

### Study subjects

This cross-sectional study involved 101,886 Nanjing residents who took part in a routine health screening at the Health Management Center of the First Affiliated Hospital of Nanjing Medical University in 2018. The participants took part in the routine physical examination once a year. A simple questionnaire was used to collect demographic characteristics and behavior factors. Abdominal venous blood was collected by vacuum anticoagulant tube or nonanticoagulant tube according to the physical examination items. Blood samples were stored at a suitable temperature and sent to the laboratory for testing within an hour. The FPG test was performed using a glucometer after an overnight or at least 8 to 12 h fast in all participants. We extracted demographic characteristics, behavioral factors, body mass index (BMI), blood glucose values, and blood pressure of the subjects from the database. To avoid a potential source of selection bias, we included all people (diagnosed with or treated for diabetes) attending the routine health screening program for the whole year.

### Definition and measurement

A fasting blood sugar level between 70 mg/dL (3.9 mmol/l) and 100 mg/dL (5.6 mmol/l) is considered normal, from 100 to 125 mg/dL (5.6 to 6.9 mmol/L) is considered prediabetes or IFG, and over 126 mg/dL (7 mmol/L) is considered diabetes [7]. IFG or prediabetes is a state higher than the normal FPG concentration but lower than the diagnostic cutoff for diabetes. GV is defined as swings in glucose levels or deviation from steady state (homeostasis).

### Statistical modeling

A univariate generalized linear model (GLM) approach was used for regression analysis and analysis of variance for FPG and FPG categories (< 5.6 mmol/L, 5.6–6.9 mmol/L, and > =7.0 mmol/L). We tested null hypotheses about the effects of various age groups and different sexes on the FPG category and investigated interactions between temporal factors and personal effects. Tukey’s test was applied for post hoc analysis if statistical significance was observed for the F test to evaluate differences among specific means. Estimated marginal means gave estimates of the predicted mean values for the cells in the model, and profile plots were used to visualize the interaction of these means. The process of model construction and analysis is described in Supplementary file 1.

Data are given as the mean and standard deviation (SD), and cross-tabulation of FPG was estimated by age, sex, height, weight, SBP, DBP, and BMI. The observed trends of FPG over days, months, and seasons were explored for interindividual variability with respect to age and sex. All analyses were performed with SPSS 25 (IBM, NY, USA), and a *P*-value < 0.05 was considered statistically significant.

### Measures of variability

Variability measures, such as variance, SD, and the coefficient of variation, have different strengths and applications. Although SD is generally preferred over variance because of its straightforward interpretation, the coefficient of variation has the power to compare data from different populations [13].

### Ethical statement

This study was approved by the ethics committee of Nanjing Medical University. The data used in this study were anonymized and irreversibly deidentified to protect participants, health care professionals, and hospital privacy.

## Results

The participants included 57,455 (56.4%) males and 44,431 (43.6%) females. Their mean ± SD age was 42.8 ± 15 years (range 12–100 years). The overall mean ± SD FPG was 5.59 ± 1.1 mmol/L, with an interquartile range of 5.0–5.7 mmol/L. The overall CV of FPG was 0.2, which implied that the SD was 20% of its mean value (Table 1).

**Table 1:** Overall variability assessment of participants’ characteristics

Variable	mean ± SD	Range	Q1	Q2	Q3	CV
Age (years)	42.8 ± 15.0	12–100	31	40	53	0.35
Weight (Kg)	66.8 ± 12.6	31–175	57	66	75	0.19
Height (cm)	166.7 ± 8.2	106–202	161	167	173	0.05
BMI (Kg/m^2^)	23.9 ± 3.8	12.6–59.2	21.5	23.7	26.0	0.16
DBP (mmHg)	75.8 ± 11.2	32–142	68	75	83	1.15
SBP (mmHg)	124.4 ± 17.6	63–263	112	123	135	0.14
FPG (mmol/L)	5.5 ± 1.1	1.1–26.3	5.0	5.3	5.7	0.20

Q1: lower quartile part; Q2: median; Q3: upper quartile part; CV: coefficient of variation

**Fig. 1:**
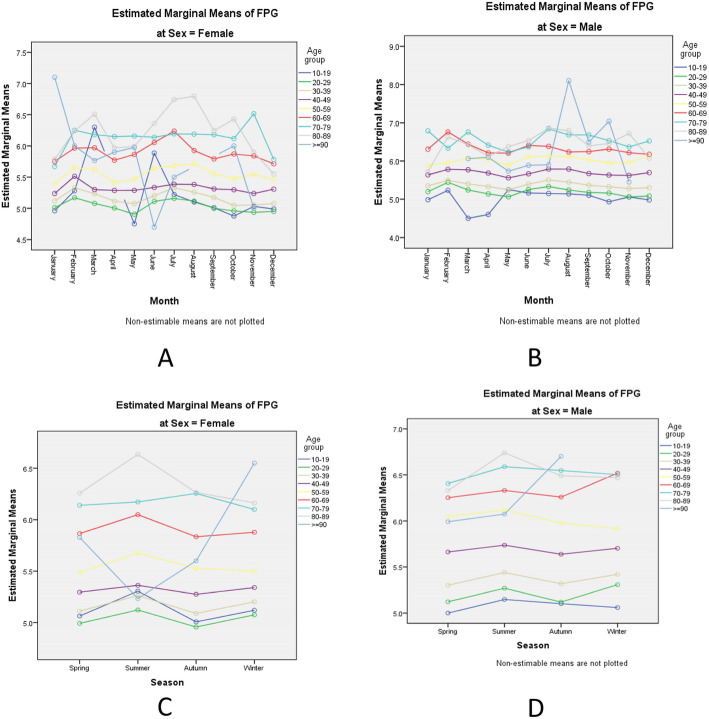
Temporal variability of fasting plasma glucose in a Chinese population

The monthly FPG variation revealed a mean value of 5.4 ± 1.07 mmol/L (range 4.3–26.3 mmol/L), with an interquartile range of 5.0–5.7 mmol/L. The seasonal FPG variation also showed a mean value of 5.2 ± 1.23 mmol/L (range 4.3–6.7), with an interquartile range of 4.8–5.5 mmol/L. FPG values for males and females were 5.7 ± 1.3 mmol/L and 5.3 ± 0.9 mmol/L, respectively. Overall, the monthly variation contributed 22% of the overall FPG variability by sex and age group (Fig. 1).

One-way ANOVA revealed that there was a significant difference in FPG among the nine age groups (*F* (8, 94,145) =1571.5, *P* < 0.001). A Tukey honest significant difference (HSD) test revealed that all nine means were significantly different from each other, although there was a slight deviation from this pattern for males aged 70–79 years and a sharp deviation for those older than 90 years in both sexes. The sex*sex mixed model revealed that the main effect of sex was significant (F (1, 94,154) =83.461, *P* < 0.05). Thus, there was an overall significant difference in FPG means between males and females. A significant main effect for age group was obtained (F (8, 94,154) =7.39, *P* < 0.05), with a good fitting effect (Eta-squared =0.15). FPG variability was higher in males than in females. The variability of FPG showed sex differences in the CV percentage difference (PD): PD =100(CV_male_ - CV_female_)/CV_female_, specifically the sex variability ratio = 34.1% more in males than females (Table 2).

**Table 2:** Descriptive characteristics of participants

Variables	Age (years)									
	10–19	20–29	30–39	40–49	50–59	60–69	70–79	80–89	> = 90	Total
**Male**	*n* = 1134	*n* = 9828	*n* = 15,119	*n* = 12,187	*n* = 10,268	*n* = 5015	*n* = 2555	*n* = 1274	*n* = 75	*n* = 57,455
Age (years)	17.9 ± 0.7	26.0 ± 2.4	34.4 ± 2.9	44.5 ± 2.9	54.0 ± 2.6	63.9 ± 2.8	74.3 ± 2.9	83.3 ± 2.6	91.8 ± 2.1	43.8 ± 15.2
Weight (kg)	70.3 ± 10.7	73.7 ± 12.2	75.6 ± 11.1	74.5 ± 9.8	73.5 ± 9.3	72.0 ± 9.4	69.2 ± 9.3	65.9 ± 9.8	62.1 ± 10.3	73.7 ± 10.7
Height (cm)	176.0 ± 4.9	174.2 ± 5.8	173.0 ± 5.8	171.2 ± 5.5	170.2 ± 5.6	169.1 ± 5.8	166.6 ± 5.4	164.7 ± 5.9	162.5 ± 5.3	171.6 ± 6.1
BMI (Kg/m^2^)	22.7 ± 3.3	24.3 ± 3.6	25.2 ± 3.3	25.4 ± 2.8	25.3 ± 2.7	25.2 ± 2.8	24.9 ± 3.0	24.3 ± 3.2	23.5 ± 3.4	25.0 ± 3.2
DBP (mmHg)	67.7 ± 6.8	74.4 ± 9.3	77.2 ± 10.4	81.1 ± 11.1	82.9 ± 10.9	81.5 ± 10.4	78.5 ± 10.8	75.4 ± 11.0	70.5 ± 11.0	78.8 ± 11.0
SBP (mmHg)	122.7 ± 10.1	123.9 ± 12.6	123.8 ± 13.8	127.2 ± 15.8	131.5 ± 16.9	136.6 ± 17.9	144.6 ± 18.6	148.1 ± 18.7	149.4 ± 18.3	128.4 ± 16.5
FPG (mmol/L)	5.1 ± 0.4	5.2 ± 0.6	5.4 ± 0.8	5.7 ± 1.2	5.7 ± 1.2	6.0 ± 1.6	6.3 ± 1.6	6.5 ± 1.7	6.2 ± 1.5	5.7 ± 1.3
**Female**	*n* = 285	n = 10,126	*n* = 13,574	*n* = 8714	*n* = 5933	*n* = 3431	*n* = 1561	*n* = 771	*n* = 36	*n* = 44,431
Age (years)	17.8 ± 0.9	25.9 ± 2.3	34.2 ± 2.8	44.5 ± 2.9	53.8 ± 2.6	63.8 ± 2.7	74.2 ± 2.8	83.1 ± 2.5	91.8 ± 2.3	41.4 ± 14.6
Weight (cm)	70.3 ± 10.7	73.7 ± 12.2	75.6 ± 11.1	74.5 ± 9.8	73.5 ± 9.3	72.0 ± 9.4	69.2 ± 9.3	65.9 ± 9.8	62.1 ± 10.3	57.4 ± 8.0
Height (Kg)	164.4 ± 4.9	161.7 ± 5.2	161.2 ± 5.2	159.7 ± 5.1	159.0 ± 5.2	157.4 ± 5.5	154.5 ± 5.2	152.0 ± 5.8	148.9 ± 8.0	160.0 ± 5.6
BMI (Kg/m^2^)	20.6 ± 2.7	21.1 ± 2.7	21.9 ± 2.8	22.9 ± 2.9	23.4 ± 2.9	24.2 ± 3.3	24.4 ± 3.3	24.2 ± 3.5	23.7 ± 3.4	22.4 ± 3.0
DBP (mmHg)	67.5 ± 7.2	69.9 ± 8.4	69.9 ± 9.2	72.3 ± 10.8	75.4 ± 10.8	76.4 ± 10.6	74.7 ± 10.6	73.5 ± 11.4	72.8 ± 11.6	71.8 ± 10.1
SBP (mmHg)	114.7 ± 11.2	112.5 ± 11.4	112.2 ± 12.7	118.0 ± 15.7	125.9 ± 17.5	135.0 ± 18.3	144.5 ± 18.3	150.3 ± 20.3	155.1 ± 22.5	118.9 ± 17.3
FPG (mmol/L)	5.1 ± 0.6	5.0 ± 0.5	5.2 ± 0.6	5.3 ± 0.8	5.5 ± 1.0	5.9 ± 1.3	6.2 ± 1.4	6.3 ± 1.6	5.8 ± 0.7	5.3 ± 0.9

We observed significant interactions for month*age*sex (F (82, 94,072) =1.89, *P* < 0.001) and day*age*sex (F (35, 94,119) =1.45, *P* < 0.05). However, there were no significant interaction effects for season*age*sex (F (23, 94,131) =1.50, *P* = 0.10) and half-year*age*sex (F (8, 94,146) =1.41, *P* = 0.19). Thus, the month and day were significantly attributed to the variability in FPG, whereas season and half-year FPG were not.

The amplitude percent mean (A % M = (highest FPG − lowest FPG)/(mean FPG) ∗ 100) for males and females was 24.7 and 24.5%, respectively. The SD percent mean, (SD % M = (SD of FPG values)/(mean FPG) ∗ 100), was higher in males (22.8%) than in females (17.0%). Examination of the multiple comparisons by age group and month was significant from January to December, except for in September (MD =0.014, 95% CI: − 0.02-0.04, *P* = 0.37).

**Fig. 2:**
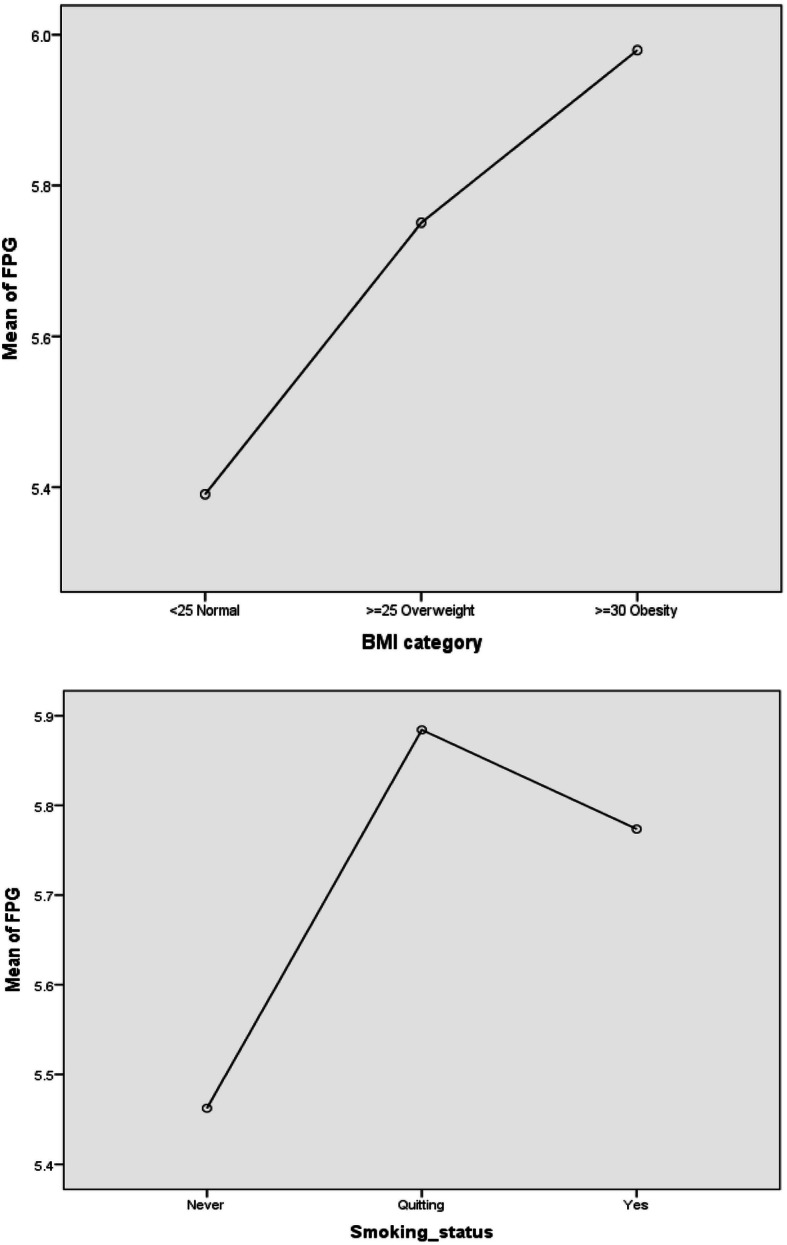
Multiple plots of temporal fasting plasma glucose variability by age and sex. **a** and **b** show the monthly distribution of the estimated marginal means of FPG by age and sex. **c** and **d** show the half-year distribution of the estimated marginal means of FPG by age and sex. **e** and **f** show the daily distribution of the estimated marginal means of FPG by age and sex. **g** and **h** show the seasonal distribution of the estimated marginal means of FPG by age and sex. FPG: fasting plasma glucose

As BMI increased, the FPG increased accordingly. The mean ± SD of FPG was 5.39 ± 0.99, 5.75 ± 1.22, and 5.9 ± 1.53 for individuals with normal weight, overweight, and obesity, respectively. The FPG mean ± SD values were as low as 5.46 ± 1.03 among those with “never” smoking status and as high as 5.88 ± 1.34 and 5.7 ± 1.52 among those with “quitting” and “yes” smoking status, respectively (Fig. 2).

Age was associated with an increased IFG for both sexes, where those aged 50–59 years recorded the highest proportion (23%). Women aged 30–39 years and men aged 40–49 years had an increased risk of type 2 diabetes (Table 3).

**Table 3:** Cross-tabulation of age group by FPG category, sex, BMI, and smoking status

Variable	Category	Age (years)								
		10–19	20–29	30–39	40–49	50–59	60–69	70–79	80–89	> = 90
**Female**	**FPG category**									
	< 5.6	236 (0.7)	7956 (25.1)	10,983 (34.7)	6434 (20.3)	3644 (11.5)	1632 (5.2)	561 (1.8)	229 (0.7)	12 (0.0)
	5.6–6.9	22 (0.3)	633 (8.4)	1561 (20.8)	1491 (19.8)	1582 (21.0)	1260 (16.8)	633 (8.4)	323 (4.3)	14 (0.2)
	> = 7.0	1 (0.1)	20 (1.7)	66 (5.6)	133 (11.2)	244 (20.5)	318 (26.7)	256 (21.5)	149 (12.5)	2 (0.2)
**Male**	**FPG category**									
	< 5.6	988 (2.9)	7521 (22.0)	11,041 (32.3)	7150 (20.9)	4694 (13.7)	1753 (5.1)	702 (2.1)	341 (1.0)	17 (0.0)
	5.6–6.9	114 (0.7)	1195 (7.6)	3176 (20.3)	3767 (24.0)	3740 (23.9)	2019 (12.9)	1099 (7.0)	531 (3.4)	23 (0.1)
	> = 7.0	0 (0.0)	49 (1.3)	239 (6.1)	722 (18.6)	1235 (31.8)	855 (22.0)	551 (14.2)	229 (5.9)	8 (0.2)
**Both**	**BMI**									
	< 25	1126 (1.6)	15,944 (23.0)	20,137 (29.0)	13,455 (19.4)	9866 (14.2)	5003 (7.2)	2456 (3.5)	1335 (1.9)	81 (0.1)
	25–29	273 (1.0)	3315 (11.7)	7309 (25.7)	6609 (23.3)	5713 (20.1)	3051 (10.7)	1477 (5.2)	621 (2.2)	28 (0.1)
	> = 30	20 (0.5)	695 (17.0)	1247 (30.5)	837 (20.5)	622 (15.2)	392 (9.6)	183 (4.5)	89 (2.2)	2 (0.0)
	**Smoking**									
	Never	1386 (1.9)	15,310 (21.4)	20,770 (29.1)	13,382 (18.7)	9799 (13.7)	5799 (8.1)	3162 (4.4)	1681 (2.4)	88 (0.1)
	Quitting	1 (0.1)	87 (5.6)	215 (13.7)	352 (22.5)	459 (29.3)	290 (18.5)	125 (8.0)	31 (2.0)	4 (0.3)
	Yes	7 (0.1)	1137 (9.2)	2778 (22.4)	3502 (28.2)	3411 (27.5)	1202 (9.7)	317 (2.6)	63 (0.5)	1 (0.0)

## Discussion

In this study, we observed significant variability in FPG by month, age, and sex. Mean glucose is commonly used to indicate whether blood sugar is high or low, as in hyperglycemia or hypoglycemia, respectively, and to determine the best action, such as to time exercise differently or use a lower medication dose. However, the variation in blood sugar levels should not be neglected. Though it is ideal to have the lowest possible average glucose, according to the Diabetes Control and Complications Trial (DCCT) and the United Kingdom Prospective Diabetes Study (UKPDS), it should be without frequent, prolonged, or severe hypoglycemia.

A follow-up of blood glucose distribution characteristics in a health examination population in Chengdu, China, from 2010 to 2016 showed higher glucose levels with increasing age, and males had higher glucose levels than females [16]. Likewise, the mean level of FPG significantly increased across age groups in both males and females. The mean level of FPG was higher in males than in females. It was evident that in the Kailuan cohort, both males and females showed a higher percentage of diabetes with increasing age, and males showed a higher percentage of diabetes than females in the same age group. Our findings were not different from those, in which the normal level of glucose was higher in the aged population than in young participants, and the percentage of males with IFG or at risk of type 2 diabetes was higher than that of females [6]. Our results add to evidence that high variability in FPG may be associated with the risk of type 2 diabetes among different age groups in a ‘healthy’ population.

The Taichung Diabetes Study measured glycemic variability by computing the annual CV of all FPG measurements in a given year and showed that the annual CV of FPG was independently associated with all-cause mortality in patients with type 2 diabetes aged 30 years and over. Participants classified based on BMI category showed that as BMI increased, FPG increased according. Patients with type 2 diabetes are generally associated with inactivity and overweight [6]. The Verona Diabetes Study found that FPG variability, as assessed by the CV of FPG for 3 years, was an independent predictor of all-cause mortality in patients with type 2 diabetes aged 56–74 years [17]. In our study, FPG variability showed sex differences in CV percentage differences, PDs/PD = 100(CVmale - CVfemale)/CVfemale), specifically, the sex variability ratio = 35.3% more in males than females, where month and day contributed to FPG variability.

A better understanding of these differences among the population is critical for implementing effective preventive measures. Participants classified based on FPG category revealed that the risks of IFG or prediabetes and diabetes were associated with maximal swings in the SD and CV. The most straightforward and easy way to measure interday variability is to calculate the SD of fasting blood glucose concentrations [18]. Siegelaar suggested SD as the easiest, best-validated, and preferred method when quantifying variability from CGM data [13]. A low SD reflected a steady glucose level with minimal swings. CV normalized glucose variability for different mean glucose levels indicated that the larger the variability is, the greater the risk of hypoglycemia.

The population density is relatively high in eastern China, and knowing the demographic transition within and across age groups and sex is key for developing public health interventions to improve overall health. To control FPG and avoid complications associated with FPG variability, such as hypoglycemia, there is a need for treatment strategies to control fluctuations in FPG variability to reduce the risk of diabetes and cardiovascular events.

This study has several limitations. First, the variability of FPG may be underestimated due to the short length of the study. Second, this study examined age- and sex-specific profile of FPG over time, but there could be other factors that might be attributed to FPG variability. Third, models other than GLM could be used in future studies. Thus, generalizing the study results should be done cautiously and in consideration of the characteristics of different populations.

## Conclusion

Temporal variability in FPG is evident in the general Chinese population and is affected by both age and sex. Interventions for optimal control of FPG variability should be directed at females and males aged 30–39 years and 40–49 years, respectively. Additionally, people should be encouraged to perform self-monitoring of fasting blood glucose and report unusual changes in blood sugar patterns for early treatment and to avoid the risk of diabetes and cardiovascular diseases.

## Supplementary Information

**Additional file 1.** Supplementary file 1. Description of statistical modeling.

## Abbreviations

ADA: American Diabetes Association
BMI: Body Mass Index
CV: Coefficient of Variation
FPG: Fasting Plasma Glucose
GLM: General Linear Model
GV: Glucose Variability
IFG: Impaired Fasting Glucose
MD: Mean Difference
SD: Standard Deviation
