# Supplementary material for: Age- and sex-specific profiles of temporal fasting plasma glucose variability in a population undergoing routine health screening
Source: BMC Public Health. 2021 Feb 9;21:320. doi: 10.1186/s12889-021-10367-x (PMC7871645; doi:10.1186/s12889-021-10367-x)
Supplement: Supplementary file 1 — Additional file 1. Supplementary file 1. Description of statistical modeling. [file 12889_2021_10367_MOESM1_ESM.docx]

**Supplementary files**

Supplementary file 1. Description of statistical modeling

**GLM**

General linear model (GLM) restricted to one dependent variable, univariate for a basic model for multiple linear regression is Y_i_=β_0_+β_1_ X_i1_+β_2_X_i2_+⋯+β_P_X_iP_+ε_i_ for each observation (i = 1, ..., n). That is n observations of one dependent variable and p independent variables. Y_i_ is the i^th^ observation of the dependent variable, X_ij_ is the i^th^ observation of the j^th^ independent variable, j =1, 2, ..., p. The values of β_j_ represent parameters to be estimated, and ε_i_ is the i^th^ independent identically distributed normal error. The GLM system of equations where the voxel time course, the beta values, and the residuals as vectors, and the set of predictors as a matrix using matrix notation:

$\left[ \begin{matrix} \begin{matrix} y_{1} \\ \vdots\end{matrix} \\ \vdots\\ y_{n} \end{matrix} \right]=\left[ \begin{matrix} \begin{matrix} 1 \\ \vdots\end{matrix} & \begin{matrix} X_{11}\ldots\\ \vdots\end{matrix} & \begin{matrix} \ldots X_{p} \\ \vdots\end{matrix} \\ \vdots& \vdots& \vdots\\ 1 & X_{n1}\ldots& \ldots X_{\mathrm{np}} \end{matrix} \right]\left[ \begin{matrix} b_{0} \\ \vdots\\ b_{p} \end{matrix} \right]+\left[ \begin{matrix} e_{i} \\ \vdots\\ e_{n} \end{matrix} \right]\Longrightarrow y=Xb+e$

Time courses of the signal, predictors, and residuals have been arranged in column form with time running from top to bottom as in the system of equations. The matrix X represents the design matrix containing the predictor time courses as column vectors. The beta values now appear in a separate vector b. The term Xb indicates matrix-vector multiplication.

**GLM diagnostics**

A good fit would be attained with beta values, b leading to predicted values $\hat{y}$, which are as close as possible to the measured value y, implying small errors:

$\hat{y}=Xb, e=y-\hat{y,} e^{'}e=\left( y-Xb \right)^{'}(y-Xb)\to min$ finds optimal beta weights minimizing the squared error values (the "least squares estimates"), b=(X'X)^-1^X'y, where X'X  matrix, corresponds to the predictor variance-covariance matrix and X'y, the scalar product which evaluates to a vector containing as many elements as predictors. Also, the variance of the measured time course can be decomposed into the sum of the variance of the predicted values (model-related variance) and the variance of the residuals: var(y)=var(y ̂ )+var(e). The square of the multiple correlation coefficient, R^2^=var(y ̂)/var(y), provides a measure of the proportion of the variance of the data, which can be explained by the model. The larger, the better.

**GLM assumptions and significance tests**

The population error values ε must have an expected value of zero at each time point, i.e. E[ε_i_] = 0, and constant variance, i.e. Var[ε_i_] = σ^2^. The error values are assumed to be uncorrelated, i.e. Cov(ε_i_, ε_j_) = 0 for all i = j, assumed to be normally distributed (e_i_ ~ N(0, σ^2^), normal i.i.d) to justify the use of t and F distributions in hypothesis tests. The multiple correlation coefficient is an important measure of the "goodness of fit" of a GLM. The R value can be transformed in an F statistic with p - 1 degrees of freedom in the numerator and n - p degrees of freedom in the denominator: F_n-1,n-p_=R^2^(n-p)/(1-R^2^)(p-1) in order to test whether a specified model significantly explains variance in a voxel time course. Comparisons between conditions can be formulated as contrasts, which are linear combinations of beta values corresponding to null hypotheses, $H_{0}: b_{i}=0.$ A t value for a specific contrast can be converted in an error probability value *P* using the equation, $t=c^{'}b/\sqrt{var(e)c'{(X'X)}^{-1}}c$ , where H_o_: c'b = 0 and H_a_: c'b = 0. If this *P* value is smaller than 0.05 and positive, the alternative hypothesis may be concluded.
